# CCNG2 Overexpression Mediated by AKT Inhibits Tumor Cell Proliferation in Human Astrocytoma Cells

**DOI:** 10.3389/fneur.2018.00255

**Published:** 2018-04-18

**Authors:** Danfeng Zhang, Chunhui Wang, Zhenxing Li, Yiming Li, Dawei Dai, Kaiwei Han, Liquan Lv, Yicheng Lu, Lijun Hou, Junyu Wang

**Affiliations:** Department of Neurosurgery, Changzheng Hospital, Second Military Medical University, Shanghai, China

**Keywords:** astrocytoma, AKT pathway, carcinogenesis, CCNG2, MK 2206

## Abstract

The cyclin family protein CCNG2 has an important inhibitory role in cancer initiation and progression, but the exact mechanism is still unknown. In this study, we examined the relationship between CCNG2 and the malignancy of astrocytomas and whether the AKT pathway, which is upregulated in astrocytomas, may inhibit CCNG2 expression. CCNG2 expression was found to be negatively associated with the pathological grade and proliferative activity of astrocytomas, as the highest expression was found in control brain tissue (*N* = 31), whereas the lowest expression was in high-grade glioma tissue (*N* = 31). Additionally, CCNG2 overexpression in glioma cell lines, T98G and U251 inhibited proliferation and arrested cells in the G0/G1 phase. Moreover, CCNG2 overexpression could increase glioma cells apoptosis. In contrast, AKT activity increased in glioma cells that had low CCNG2 expression. Expression of CCNG2 was higher in cells treated with the AKT kinase inhibitor MK-2206 indicating that the presence of phosphorylated AKT may inhibit the expression of CCNG2. Inhibition of AKT also led to decreased colony formation in T98G and U251 cells and knocked down of CCNG2 reversed the result. Finally, overexpression of CCNG2 in glioma cells reduced tumor volume in a murine model. To conclude, low expression of CCNG2 correlated with the severity astrocytoma and CCNG2 overexpression could induce apoptosis and inhibit proliferation. Inhibition of AKT activity increased the expression of CCNG2. The present study highlights the regulatory consequences of CCNG2 expression and AKT activity in astrocytoma tumorigenesis and the potential use of CCNG2 in anticancer treatment.

## Introduction

Astrocytomas are the most prevalent of all primary gliomas and thought to originate from astrocytes located in the cerebrum (1, 2). Tumors of the central nervous system were first classified according to a pathological diagnostic standard by the World Health Organization (WHO): grade I, pilocytic astrocytomas; grade II, diffuse astrocytomas; grade III, anaplastic astrocytomas; and grade IV, glioblastomas (GBMs) (3). Pilocytic and diffuse astrocytomas are more often found in children or young adults, whereas anaplastic astrocytomas and GBMs are more prevalent in adults. Approximately, 65% of astrocytomas are highly aggressive grade IV GBMs from which most patients die within the first year of diagnosis (4, 5). The 5-year survival rate is less than 5% even with optimal multimodality therapeutics, which includes maximal surgical resection, radiotherapy, and chemotherapy (6).

Management of gliomas is especially hampered by the ability of certain cells within neoplasms to survive under surgery and therapy to form recurrent therapy-resistant lesions (7). To understand the mechanism of these cells, genomic comparative analysis has further classified astrocytomas into subtypes (8, 9). Poor survival has been linked to AKT signaling, indicative of proliferation or of angiogenesis, and mesenchyme subtypes, whereas a more favorable prognosis is linked to Notch signaling (10). AKT is known to be activated by alterations in the expression of the epidermal growth factor (*EGFR*) and phosphatase and tensin homolog (*PTEN*) genes, which are also associated with the aggressiveness of tumors (11). Increased expression of *EGFR* and decreased expression of *PTEN* are linked to a poor prognosis. It is thought that a higher level of *PTEN* may influence Notch signaling and promote a more differentiated type of tumor leading to improved prognosis (12).

AKT phosphorylation regulates the transcriptional activity of proteins involved in cyclin expression (13). Cyclins are a family of proteins that are known to be expressed and degraded during progression through the cell cycle and are associated with the severity of astrocytoma grade (14, 15). Although, the nucleotide sequence of cyclin G1 and cyclin G2 (CCNG2) are similar, cyclin G2 contains a C-terminal PEST region suggesting that CCNG2 degradation may be regulated in cell cycle progression (16). CCNG2 expression is significantly higher in cycle-arrested and terminally differentiated cells (16, 17). Furthermore, in a recent study the PEST region of CCNG2 has been shown to have a pivotal role in EGFR-associated degradation (18). Several studies indicate that CCNG2 may have an inhibitory role in the progression of cancer as lower expression of CCNG2 is often found in more aggressive cancers and is associated with lower overall survival (19–21). Therefore, *CCNG2* is often proposed to be a tumor suppressor gene through its regulation of cell proliferation.

In this study, we investigate CCNG2 expression and its inhibitory function in surgical samples and human astrocytoma cells. We also assess possible interactions between AKT-mediated regulation and CCNG2. We found that increased CCNG2 expression could inhibit proliferation, induce G0/G1 phase arrest, and promote apoptosis in glioma cells *in vitro* and that levels of CCNG2 are mediated by AKT.

## Materials and Methods

### Tumor Samples and Cell Culture

The current study included 93 patients who attended our institute from 2014 to 2015. Overall, 31 high-grade astrocytomas (WHO grade III–IV), 31 low-grade astrocytomas (WHO grade I–II), and 31 paratumor tissue samples were collected *via* surgical resection. The gliomas were graded in accordance with the WHO pathological diagnostic standard (3). Paratumor tissues were taken from peripheral nontumor glial brain tissue from patients. The clinicopathological features of patients included are detailed in Table 1. Samples were divided and either frozen in liquid nitrogen and stored at −80°C or kept in RNAlater (Ambion, Austin, TX, USA) at −20°C. The study was conducted in accordance with the Declaration of Helsinki and approved by the Institutional Review Board of Shanghai Second Military Medical University. Informed consent was returned from all patients included in the current study or their direct relatives.

**Table 1:** Relationships between CCNG2 expression in human glioma tissues and clinicopathological features.

Clinicopathological features	No. of cases	CCNG2 (*n*, %)		*p*Value
		High	Low	
**Gender**				
Male	39	17 (44)	22 (56)	>0.05
Female	23	10 (43)	13 (57)	
**Age**				
<45	26	11 (44)	15 (44)	>0.05
≥45	36	16 (44)	20 (56)	
**WHO grade**				
I	12	8 (67)	4 (33)	<0.05
II	19	13 (68)	6 (32)	
III	17	3 (18)	14 (82)	
IV	14	3 (21)	11 (79)	

*WHO, World Health Organization*.

Glioma cell lines, such as T98G, U138, U251, and A172, were obtained from the American Type Culture Collection and cultured at 37°C in a humidified atmosphere of 5% CO_2_ in a ratio 1:1 mixture of Dulbecco modified Eagle medium (DMEM; HyClone, Lanzhou, China) and supplemented with 10% FBS, 100 IU/mL penicillin, and 100 µg/mL streptomycin, all of which were purchased from Life Technologies (Carlsbad, CA, USA).

### Regents and Antibodies

MK-2206 was provided by Merck and Co., Inc. (Kenilworth, NJ, USA). MK-2206 was dissolved in DMSO for *in vitro* experiments. Antibodies for western blotting, including β-actin, CCNG2, P-gp, MRP1, caspase-3, BCL-2, MMP2, and MMP9, were all purchased from Abcam (Cambridge, UK), phospho-AKT and total-AKT were all purchased from Cell Signaling Technology (Danvers, MA, USA) (all 1:1,000 dilutions).

### Immunohistochemistry

Immunohistochemical staining was performed using a method described previously (22). Briefly, thawed samples were fixed in 4% formalin and embedded in paraffin for histopathological analysis. Samples were deparaffinized with xylol and then sliced into 4 µm sections. Sections were rehydrated using a graded ethanol series. A heat-induced epitope protocol was used for antigen-retrieval (95°C for 40 min). Samples were incubated in methanol containing 0.3% hydrogen peroxide to block endogenous peroxidase. Samples were blocked with protein serum (Vectastain Elite ABC kit; Vector Laboratories, Inc., Burlingame, CA, USA) and then incubated (overnight at 4°C) with polyclonal rabbit anti-human CCNG2 or Ki67 antibody at 1:1,000 (MBL International Corporation, Nagoya, Japan). After washing three times in TBST (150 mM NaCl, 10 mM Tris–HCl, pH 7.6), sections were incubated with secondary antibody for 20 min at room temperature. Peroxidase-conjugated biotin-streptavidin complex (Dako, Glostrup, Denmark) was then applied to the sections for 20 min. Sections were visualized with 3, 3′-diaminobenzidine and counterstained with hematoxylin. The negative control used nonimmune serum instead of primary antibody.

### Quantitative PCR Analysis

Total RNA was extracted using TRIzol reagent (Life Technologies) following manufacturer’s instructions. RNA was reverse-transcribed to cDNA using Super-Script First-Strand cDNA System (Invitrogen, Carlsbad, CA, USA), and amplified with Platinum SYBR Green qPCR SuperMix-UDG (Invitrogen), forward and reverse primers, and template cDNA (10 ng). The reaction conditions were 95°C for 5 min, and then 32 cycles of 95°C for 15 s, 60°C for 1 min, and 72°C for 30 s. β-actin was used as an internal control. The relative expression of mRNA was quantified using the −2^ΔΔCT^ method (23).

### Protein Isolation and Western Blot Analysis

Proteins were extracted with RIPA buffer. Protein concentration was determined using a bicinchoninic acid protein assay. Approximately 30 µg of protein from each sample was separated on a 10% SDS-polyacrylamide gel and transferred to polyvinylidene difluoride membranes. Membranes were blocked with 5% skim milk in TBST and incubated with primary antibodies overnight at 4°C followed by incubation with the corresponding secondary antibodies for 1 h at room temperature. Proteins were detected on membranes, after washing in TBST, using Super ECL Plus Detection Reagent (Thermo Fisher Scientific, Carlsbad, CA, USA).

### Constructs and Transfection

Glioma cells were grown to 55–65% confluency without antibiotics on 35 mm tissue culture plates prior to transfection. Human CCNG2 cDNA was inserted between the HindIII and Apa1 sites of a pcDNA3.1 mammalian expression vector (Life Technologies). Purified plasmid DNA was first mixed with Lipofectamine 2000 (Thermo Fisher Scientific) and then added to the culture dish (2 µg DNA/culture dish). The transfection procedure was carried out following the manufacturer’s instructions (Thermo Fisher Scientific). Twelve hours post-transfection, the media were discarded and replaced with fresh media without antibiotics. CCNG2 expression in glioma cells was verified by both western blotting and qRT-PCR 48 h after transfection.

### Cell Proliferation and Apoptosis Assay

Cell proliferation was determined by a 3-(4,5-dimethylthiazol-2-yl)-2,5-diphenyltetrazolium bromide (MTT) assay as previously described (24). The MTT absorbance was examined at 570 nm in a SpectraMax Plus 384 Microplate Reader (Molecular Devices, LLC, USA) at 24, 48, 72, 96, and 120 h after treatment. The apoptosis rate of cells was assessed using a MEBCYTO Apoptosis Kit (Medical and Biological Laboratories Co., Ltd., Aichi, Japan) according to manufacturer’s instructions. Briefly, 1 × 10^5^ cells were stained with Annexin V-fluorescein isothiocyanate and propidium iodide (PI) for 25 min after trypsinization and then analyzed with flow cytometry (BD FACSCalibur, Becton Dickinson, San Jose, CA, USA). The apoptosis rate was determined as the ratio of Annexin V-positive apoptotic cells in all counted cells.

### Colony Formation Assay

Cell proliferation was assessed by a soft agar colony formation assay. A 6-well plate containing a 1.5 mL bottom layer and 0.5 mL top layer of agar was used (5.1 mg/mL, Difco Laboratories, Detroit, MI, USA). Cells (10^4^/well) were transferred onto the bottom layer and then overlaid with a top layer and cultured at 37°C in 5% CO_2_. Giemsa staining was used to quantify the formation of colonies on the seventh day.

### Cell Cycle Assay

Cells were first collected from the medium by trypsinization, washed and suspended in cold PBS, and centrifuged at 1,000 rpm for 5 min. Cells were then fixed in 4.5 mL 70% cold ethanol at 4°C. After fixation, cells were cultured in 0.2 mg/mL PI including RNase A (1 mg/mL, Sigma) and 0.1% Triton X-100 for 30 min. The cell cycle distribution was determined by fluorescence-activated cell sorting (FACS) analysis (FACSCalibur, BD Biosciences, San Diego, CA, USA).

### Tumor Xenograft Model and Tumorigenicity Assay

A xenograft tumor model was generated using 6-week-old male BALB/c nude mice (*n* = 20) obtained from Shanghai Laboratory Animal Company (SLAC, Shanghai, China). U251 cells were transfected with an empty vector or CCNG2 and then resuspended in PBS. Mice were randomly divided into four groups of five mice. Each mouse was inoculated with 6 × 10^6^ cells in 100 µL of PBS subcutaneously into the left armpit. Tumor diameters were measured with a vernier caliper every 7 days and tumor volumes were calculated using the following equation: tumor volume = 1/2 (length × width^2^). After 5 weeks, mice were sacrificed to retrieve tumors. All experiments were approved and performed according to the guidelines of the Ethics Committee of the Institutional Review Board of Shanghai Second Military Medical University (Shanghai, China), conformed to the Principles of Laboratory Animal Care (National Society for Medical Research), and were conducted according to the National Institutes of Health guidelines.

### Statistics

Data from three replicate tests in this study were expressed as mean ± SD. The Student’s *t*-test and analysis of variance were used to compare differences between two and three (or more) groups, respectively. ImageJ software (NIH, USA) was used for the visualization and quantification of immunoblot bands. The significant threshold was determined as *P* < 0.05.

## Results

### CCNG2 Is Down-Regulated in Astrocytoma Tissue and Cells

**Figure 1:**
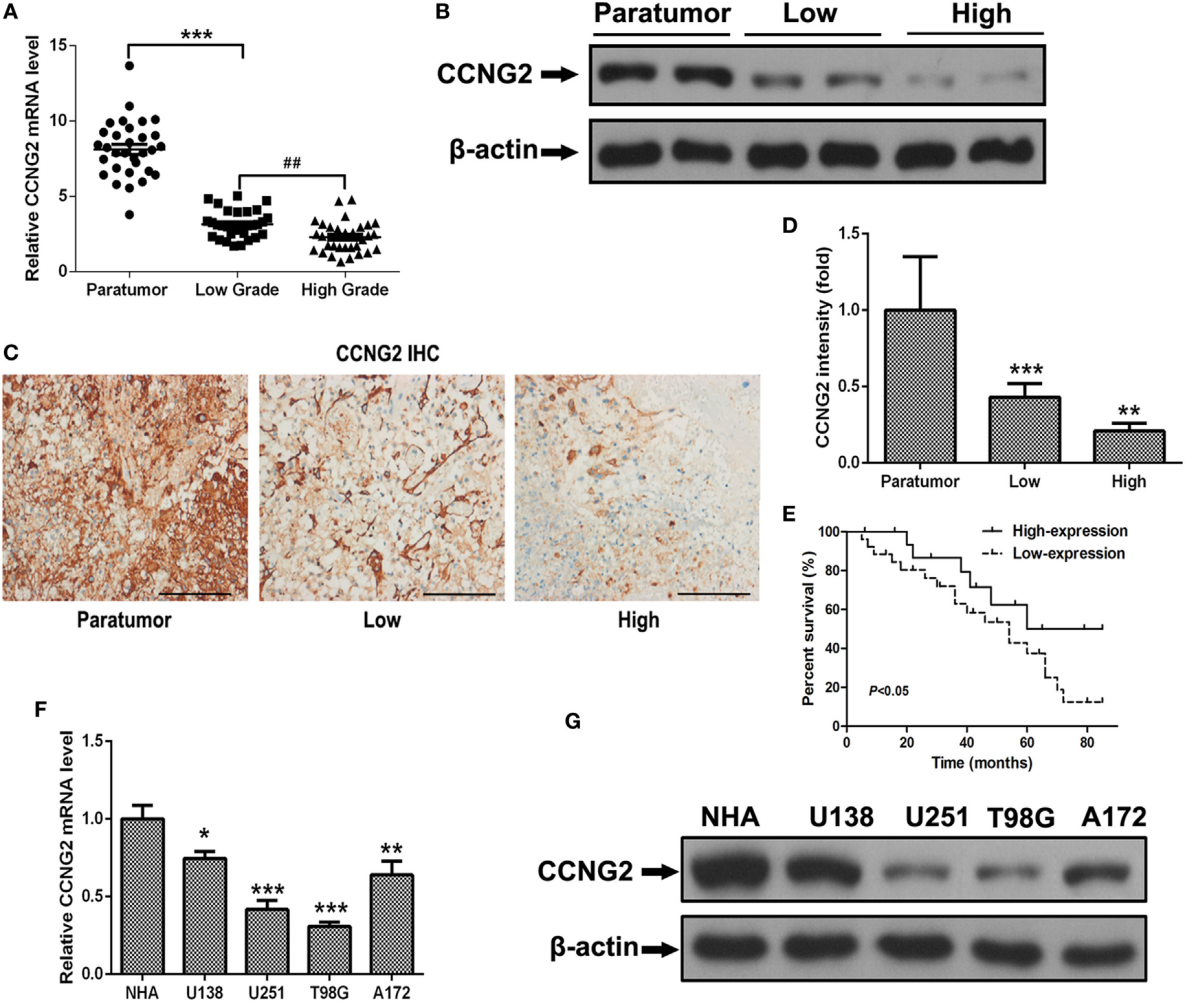
CCNG2 expression of patients with astrocytoma and glioma cell lines. **(A,B)** CCNG2 mRNA and protein levels were detected by RT-PCR and western blotting in low- and high-grade astrocytoma surgical samples (*n* = 31) compared with paratumor compared samples (*n* = 31). **(C,D)** Expression of CCNG2 was determined by immunohistochemistry staining in all tissues and quantified. Scale bars: 100 µm. **(D)** Kaplan–Meier survival curve of patients with low- and high expression of CCNG2. **(E)** CCNG2 expression levels in four glioblastoma cell lines and normal astrocyte cells (*n* = 3) were analyzed by RT-PCR and western blotting. **(F,G)** CCNG2 mRNA and protein levels were detected by RT-PCR and western blotting in four cell lines (U138, U251, A172, and T98G).*P < 0.05, ***P < 0.001, ^###^*P* < 0.001 by unpaired two-tailed Student’s *t*-test.

To determine whether the expression of CCNG2 was influenced by the grade of astrocytoma, we assessed CCNG2 mRNA expression and protein levels in paratumor compared samples to low- and high-grade astrocytoma tissue (Figure 1A). CCNG2 expression correlated with the pathological grade of astrocytoma, with the highest level of expression found in paratumor compared samples (*n* = 31) and the lowest expression found in high-grade astrocytomas (*n* = 31, *P* < 0.001). The CCNG2 level in the low-grade astrocytoma (*n* = 31) was also higher than that of the high-grade astrocytomas (*P* < 0.01). These findings were consistent with western blot analysis of CCNG2 protein levels (Figure 1B). Expression of CCNG2 was also determined by immunohistochemistry in all tissues. Paratumor compared samples sections were stained more intensely with 3,3′-diaminobenzidine indicating a higher presence of CCNG2 than in low and high grades of astrocytoma (Figures 1C,D). Kaplan–Meier survival curve suggested that the expression of CCNG2 was reflected in patient survival rates with longer survival associated with a higher expression of CCNG2 (Figure 1E). We found that CCNG2 was high expression in 67% (8/12) of WHO I, 68% (13/19) of WHO II gliomas, 18% (3/17) of WHO III, and 21% (3/14) of WHO IV gliomas, indicating a loss of CCNG2 in 81% of high-grade gliomas (Table 1). Thus, these findings indicate the dysregulation and progressive loss of CCNG2 expression in gliomas carcinogenesis and development.

Finally, the relationships between CCNG2 expression and clinicopathological characteristics of astrocytomas were analyzed. CCNG2 expression was related to advanced pathological grade (*P* < 0.01). No statistically significant relationships between age and gender and CCNG2 expression were detected. Wilcoxon rank sum test indicated that CCNG2 expression and pathological grade were independently correlated with prognosis of astrocytomas (*p* < 0.05), while no significant relationships were detected between survival time and age and gender (Table 2).

**Table 2:** Wilcoxon rank sum test of the relationship between CCNG2 expression in human glioma tissues and clinicopathological features.

	*B*	SE	Wald	df	Sig.	Exp(*B*)	95% CI for Exp (*B*)	
							Lower	Upper
WHO grade	0.780	0.186	17.593	1	0.000	2.181	1.515	3.139
CCNG2	0.964	0.398	5.863	1	0.015	2.621	1.202	5.718
Age	0.437	0.321	1.857	1	0.173	1.548	0.826	2.901
Gender	0.258	0.192	1.804	1	0.179	1.295	0.888	1.888

### Overexpression of CCNG2 Inhibits Cell Proliferation in Human Primary Glioblastoma Cell Lines

**Figure 2:**
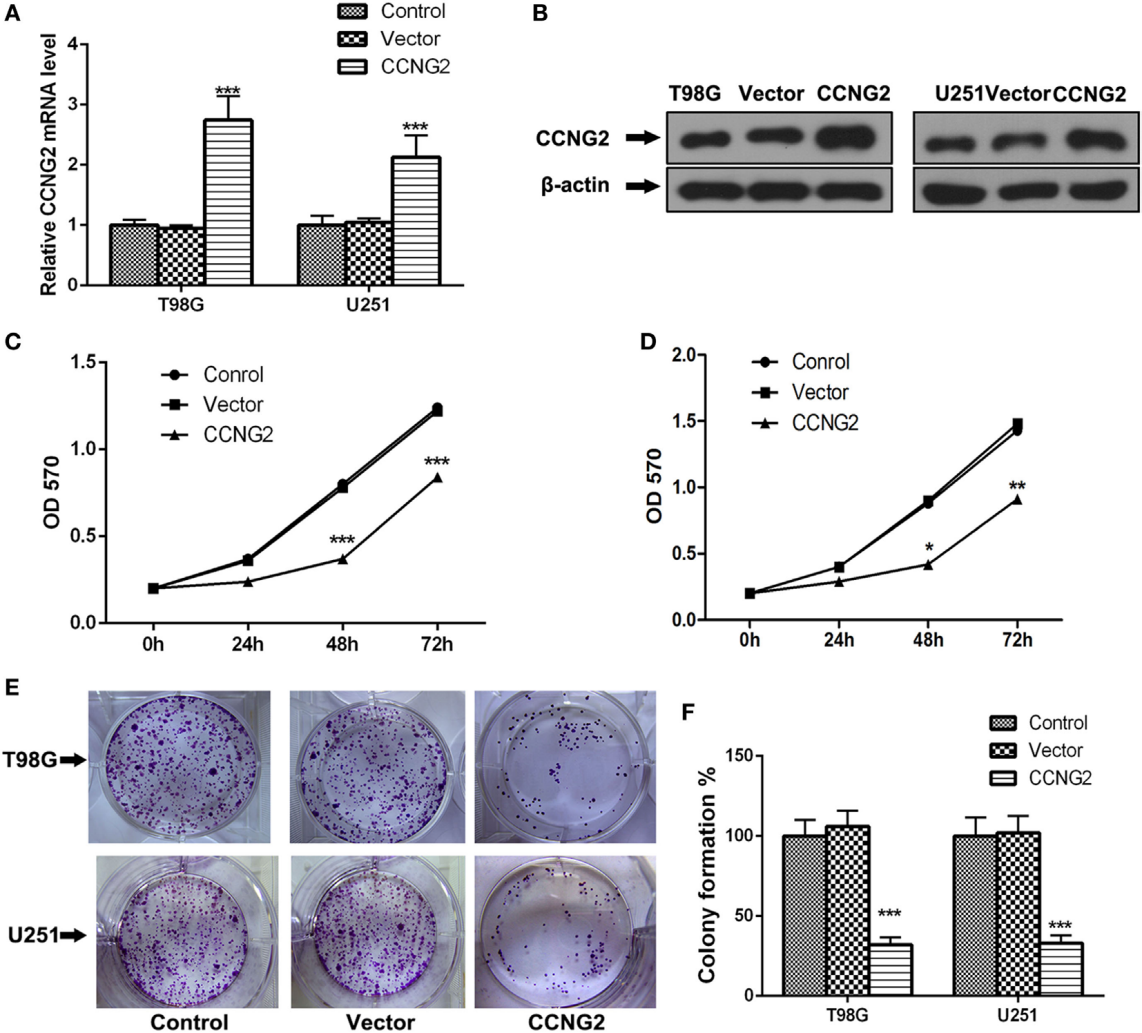
Overexpression of CCNG2 inhibits cell proliferation in T98G and U251 cells. **(A,B)** RT-PCR and western blot detection of CCNG2 expression levels in T98G and U251 control cells or cells transfected with CCNG2 or vector. Cell proliferation was detected by MTT assay in **(C)** T98G and **(D)** U251 control cells or cells transfected with CCNG2 or vector. **(E,F)** Colony formation assays in T98G and U251 cells showed that CCNG2 overexpression resulted in a significant decrease in colony number compared with the control groups. Data were expressed as means ± SEM (*n* = 3).*P < 0.05, **P < 0.01, ***P < 0.001 by unpaired two-tailed Student’s *t*-test.

Expression and protein level of CCNG2 were also assessed in glioblastoma cell lines, U138, U251, T98G, and A172. Significantly lower levels of CCNG2 expression were found in T98G and U251 compared to normal astrocytes and the other cell lines (Figures 1F,G). The T98G and U251 cell lines were subjected to further analysis. CCNG2 was overexpressed in the T98G and U251 cell lines to assess the impact on cell division. RT-PCR and western blot were used to detect expression levels of CCNG2 in T98G and U251 cells transfected with CCNG2 or empty vector (Figures 2A,B). Cell proliferation was detected by MTT assay in T98G and U251 and found to be reduced in the cell lines overexpressing CCNG2 (Figures 2C,D). Colony formation assays in T98G and U251 cells showed that CCNG2 overexpression resulted in a significant decrease in colony number compared with the control groups (Figures 2E,F).

### CCNG2 Expression Influences Cell Apoptosis and Cell Cycle Progression in Glioblastoma Cell Lines

**Figure 3:**
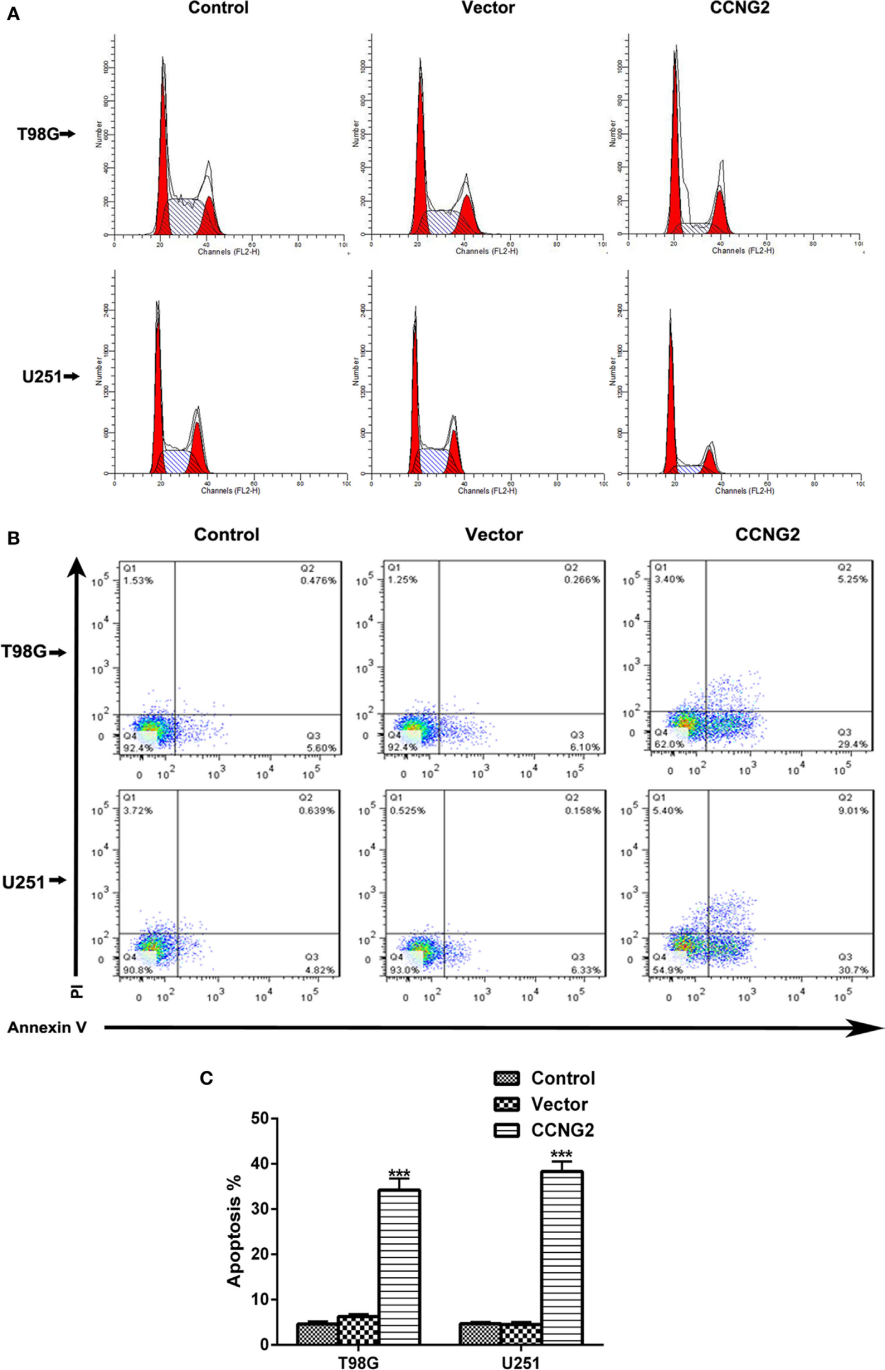
Effects of CCNG2 overexpression on cell apoptosis and cell cycle progression. **(A)** Propidium iodide staining and flow cytometry were used to examine the effect of CCNG2 overexpression on cell cycle progression in T98G and U251 cells. The quantitative results are summarized in Tables 3 and 4. **(B,C)** Analysis of apoptosis rates by flow cytometry in T98G and U251 control cells or cells transfected with CCNG2 or vector. Data were expressed as means ± SEM (*n* = 3). ***P < 0.001 by the unpaired two-tailed Student’s *t*-test.

Propidium iodide staining and flow cytometry were used to assess whether overexpressing of CCNG2 in T98G and U251 glioma cells influenced cell apoptosis and cell cycle progression. The quantitative results are summarized in Tables 3 and 4. The fraction of cells in G0/G1, S, and G2/M phases were counted (Figure 3A). The overexpression of CCNG2 caused a significantly higher fraction of glioma cells in the G0/G1 phase (56.9% in U251 cells and 33.5% in T98G cells) than in the non-transfected cells (33.9% in U251 cells and 17.6% in T98G cells), whereas cells in S phase were reduced by around 50%, suggesting that CCNG2 expression might induce a G0/G1 phase arrest in U251 and T98G cells. Flow cytometry analysis of apoptosis rates of T98G and U251 cells transfected with CCNG2, vector, or control are shown in Figure 3B. The percentage of apoptotic cells increased significantly in both cell lines that were overexpressing CCNG2 (Figure 3C).

**Table 3:** Distribution of the cell cycle in the T98G cell line after transfection.

Cell cycle	Group (mean ± SD, %)		
	Control	Vector	CCNG2
G0/G1	33.58 ± 1.44	36.95 ± 1.23	56.72 ± 1.57
S	49.42 ± 1.14	44.51 ± 1.85	22.73 ± 0.75
G2/M	16.82 ± 0.54	19.05 ± 0.68	24.54 ± 0.72

**Table 4:** Distribution of the cell cycle in the U251 cell line after transfection.

Cell cycle	Group (mean ± SD, %)		
	Control	Empty vector	CCNG2
G0/G1	38.70 ± 1.47	39.88 ± 1.16	60.49 ± 1.37
S	37.67 ± 1.35	40.86 ± 1.14	21.73 ± 0.92
G2/M	23.63 ± 1.07	19.26 ± 0.76	17.77 ± 0.85

### Effects of CCNG2 Overexpression on Cell Proliferation *In Vitro*

**Figure 4:**
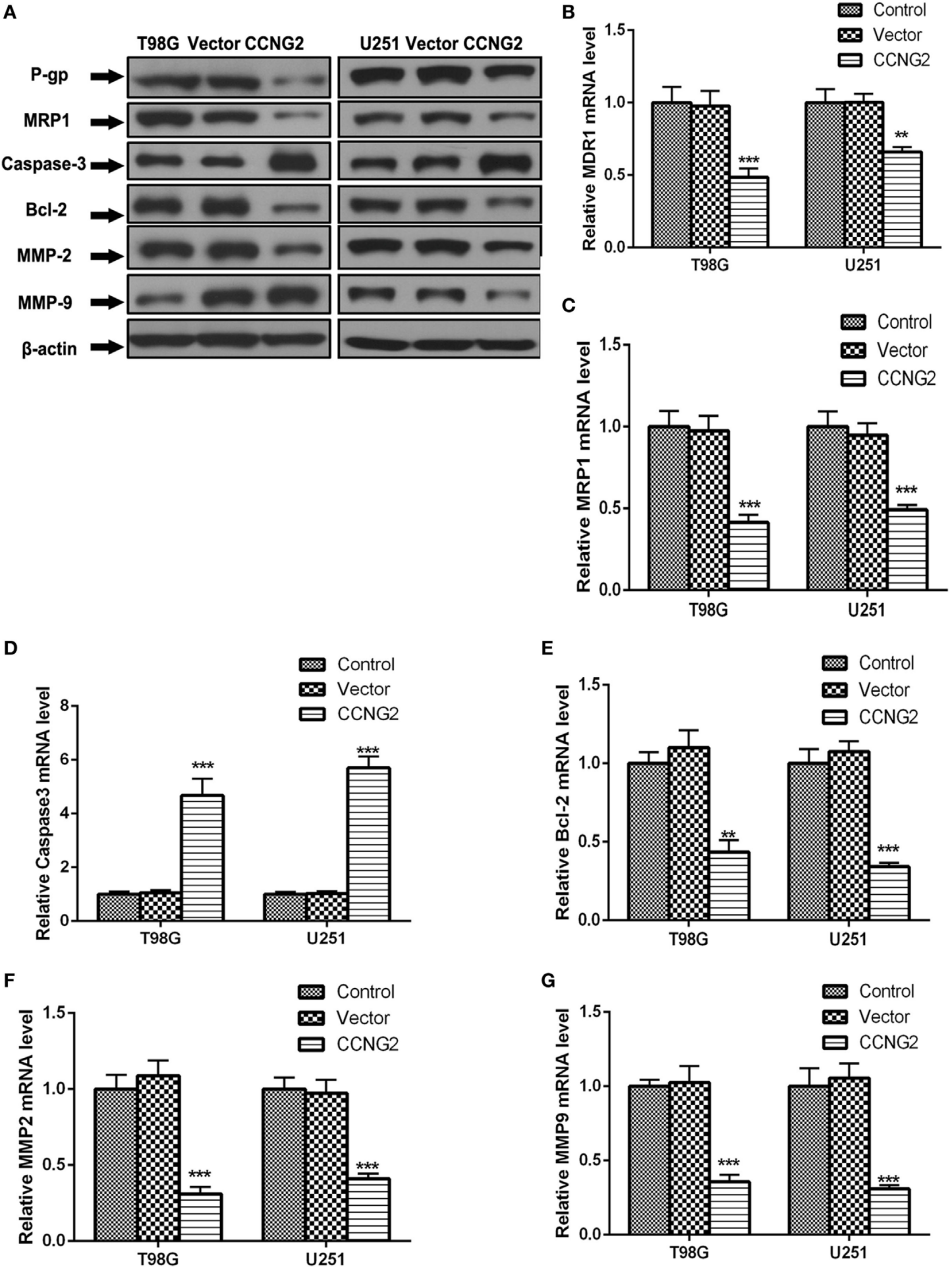
Effect of overexpressed CCNG2 on protein and mRNA expression of MDR1 (P-gp), MRP1, Bcl-2, caspase-3, MMP-2, and MMP-9 in T98G and U251 cells. **(A–G)** Western blot and RT-PCR analysis the protein and mRNA levels of MDR1 (P-gp), MRP1, caspase-3, Bcl-2, MMP-2, and MMP-9 in T98G and U251 cells transfected with CCNG2 or vector or control. Data were expressed as mean ± SEM (*n* = 3). **P < 0.01 and ***P < 0.001.

Recombinant CCNG2 lentivirus and empty lentiviral vector with GFP were successfully transfected into U251 and T98G cells to obtain overexpressed CCNG2 U251 and T98G sublines (Lenti-CCNG2 cells) and empty vector-transfected cells (Lenti-GFP cells) (25). The effect of overexpressed CCNG2 on protein level and mRNA expression of the drug-resistant proteins MDR1 (P-gp) and MRP1, the apoptosis-related proteins Bcl-2 and caspase-3, and extracellular matrix proteins MMP-2, and MMP-9 were determined in T98G and U251 cells (Figure 4). Western blot analysis indicated that MDR1 (P-gp), MRP1, Bcl-2, MMP-2, and MMP-9 protein levels were reduced in both T98G and U251 cells transfected with CCNG2, whereas levels of caspase-3 were increased (Figure 4A). Caspase-3 is required for cell apoptosis and normal brain development (26). An increase in caspase-3 activity indicates increased apoptosis when CCNG2 is overexpressed in the glioma cell lines and Bcl-2 is downregulated. The number of apoptotic cells in the CCNG2 overexpression group was greater than that of the blank and negative control group. These findings revealed that CCNG2 overexpression may induce apoptosis by activating caspase-3 and downregulating Bcl-2, MDR1, and MRP1 in glioma cells. The effect of overexpressing CCNG2 was confirmed by mRNA analysis (Figures 4B–G). RT-PCR confirmed that mRNA levels of MDR1 (P-gp), MRP1, Bcl-2, MMP-2, and MMP-9 were significantly reduced in T98G and U251 cells transfected with CCNG2 whereas caspase-3 was significantly increased.

### Inhibition of AKT Prevents Cell Proliferation in T98G and U251 Glioma Cells

**Figure 5:**
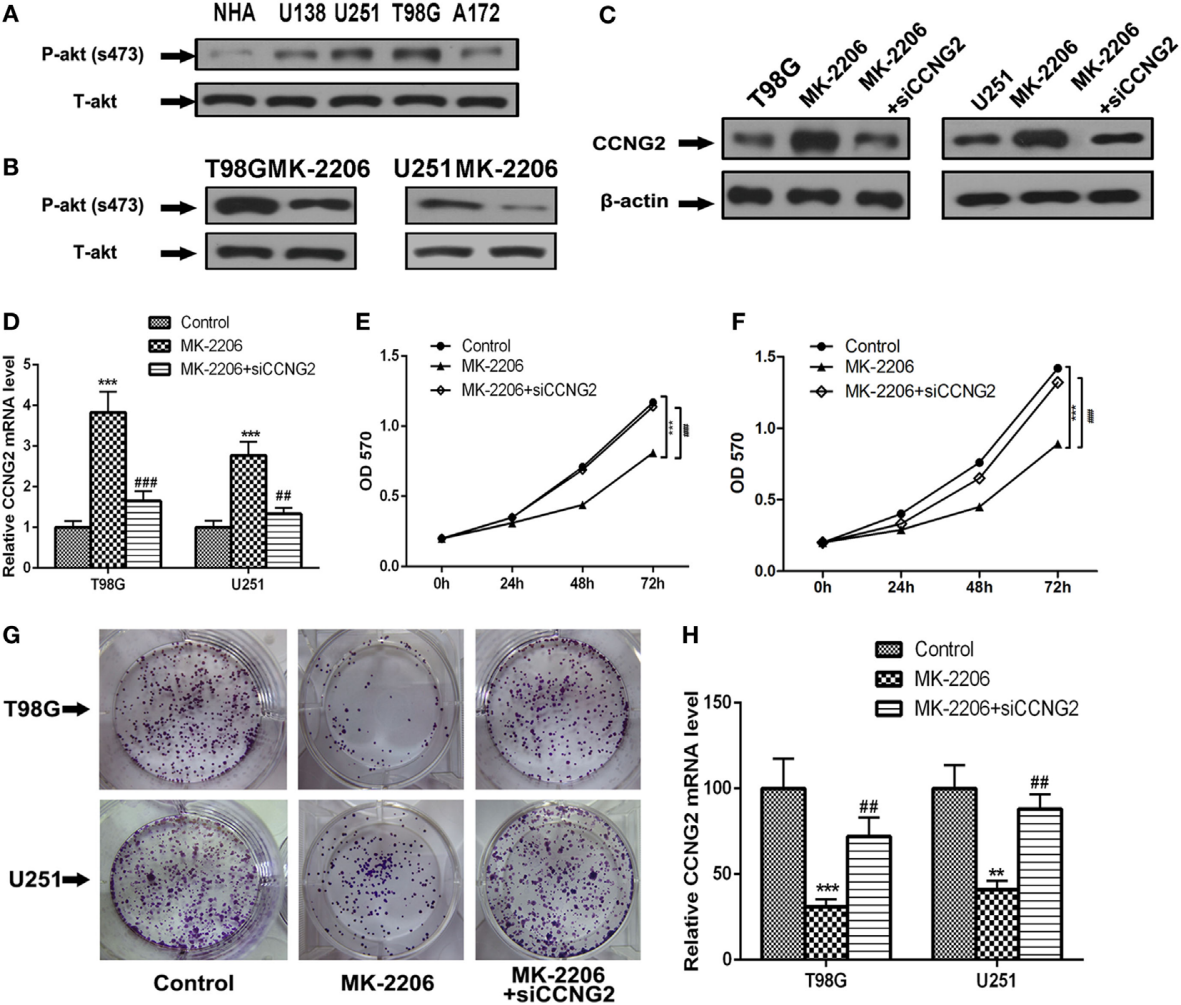
Inhibition of AKT inhibits cell proliferation in T98G and U251 cells is mediated by the modulation of CCNG2. **(A)** Phospho-AKT (S437) and total-AKT protein expression levels in four glioblastoma cell lines and normal astrocytes cell were analyzed by western blotting. **(B)** Western blotting detected the expression levels of phospho-AKT (S437) and total-AKT in T98G and U251 cells treated with AKT kinase inhibitor MK-2206 or a vehicle control. **(C,D)** RT-PCR and western blotting detected the expression levels of CCNG2 in T98G and U251 cells treated with MK-2206 or siCCNG2 or a vehicle control. Cell proliferation was detected by MTT assay in **(E)** T98G and **(F)** U251 cells treated with MK-2206 or siCCNG2 or a vehicle control. **(G,H)** Colony formation assays in T98G and U251 cells showed that inhibited AKT resulted in a significant decrease in colony number compared with the vehicle control groups and siCCNG2 restored this suppression. Data were expressed as means ± SEM (*n* = 3). **P < 0.01, ***P < 0.001, ^##^*P* < 0.01, ^###^*P* < 0.001 by the unpaired two-tailed Student’s *t*-test.

To determine if AKT could be implicated in regulating cell proliferation in astrocytoma we assessed AKT activity in glioma cell lines compared with normal astrocytes (Figure 5A). The level of phosphorylated AKT was increased in most of the cell lines, especially in T98G and U251, which also had the lowest levels of CCNG2. We also detected levels of phospho-AKT (S437) and total-AKT in T98G and U251 cells treated with the AKT kinase inhibitor MK-2206 (Figure 5B). Not only did MK-2206 reduce the activity of AKT in T98G and U251 cells, but it also increased levels of CCNG2 protein and mRNA expression while CCNG2 knockdown reversed the levels (Figures 5C,D). Furthermore, cell proliferation detected by an MTT assay was found to be reduced in T98G and U251 cells treated with MK-2206 (Figures 5E,F) and inhibition of AKT also led to a significant decrease in colony formation in T98G and U251 cells and CCNG2 knockdown could increase this inhibition (Figures 5G,H).

**Figure 6:**
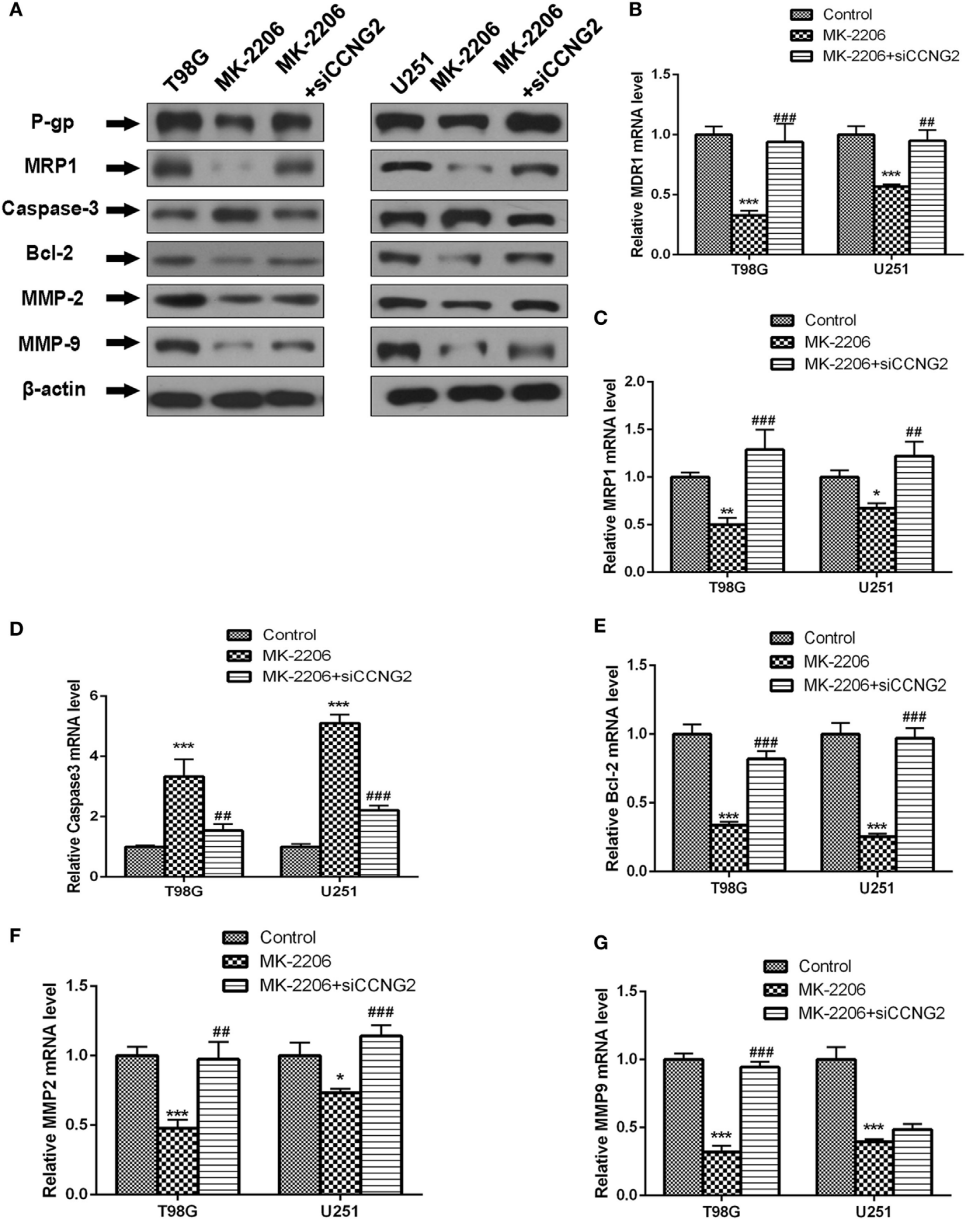
Effect of inhibited AKT on protein and mRNA expression of MDR1 (P-gp), MRP1, Bcl-2, caspase-3, MMP-2, and MMP-9 in T98G and U251 cells. **(A–G)** Western blot and RT-PCR analysis of protein and mRNA levels of MDR1 (P-gp), MRP1, caspase-3, Bcl-2, MMP-2, and MMP-9 in T98G and U251 cells treated with MK-2206 or siCCNG2 or vehicle control. Data were expressed as mean ± SEM (*n* = 3). **P < 0.01 and ***P < 0.001, ^#^*P* < 0.05, ^##^*P* < 0.01, ^###^*P* < 0.001.

We also determined protein and mRNA levels of MDR1 (P-gp), MRP1, Bcl-2, caspase-3, MMP-2, and MMP-9 in T98G and U251 cells treated with MK-2206 and transfected with siCCNG2 (Figure 6). A similar pattern to CCNG2 overexpression was observed when AKT was inhibited. All protein mRNA levels were significantly reduced apart from caspase-3, which was significantly increased while siCCNG2 reversed the levels.

### CCNG2 *In Vivo* Tumor Formation From U251 Cells in Nude Mice

**Figure 7:**
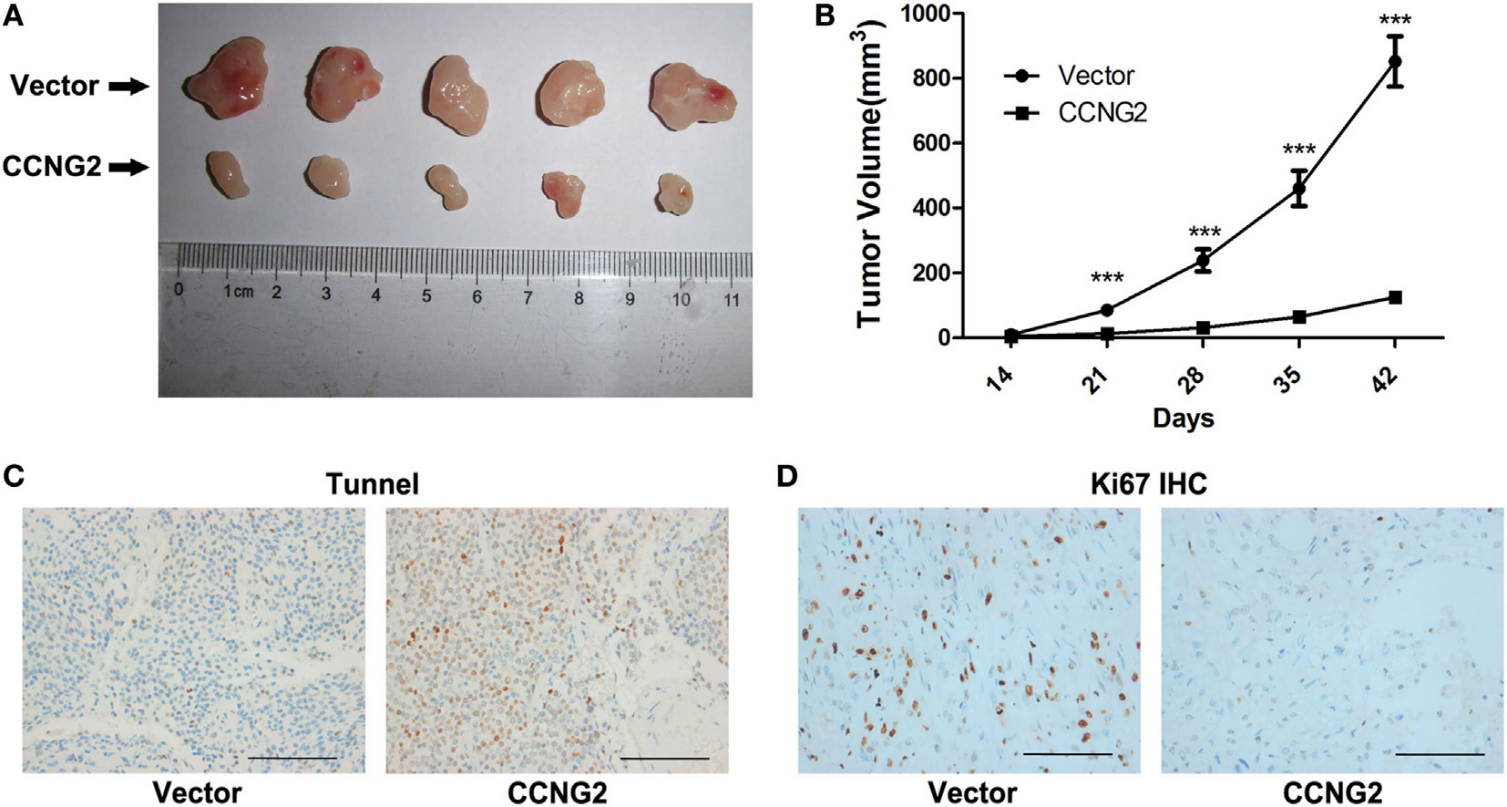
CCNG2 *in vivo* tumor formation from U251 cells in nude mice. Stable CCNG2 transfected and empty vector U251 cells were implanted subcutaneously in the left flank of nude mice. **(A)** After 42 days, mice were sacrificed and tumors were excised. **(B)** Effect of CCNG2 on tumor volume in a nude mice model. Apoptosis and Ki67 expression were detected by **(C)** TUNEL and **(D)** immunohistochemistry assays. Scale bars: 100 µm. ***P < 0.001 by the unpaired two-tailed Student’s *t*-test.

U251 cells transfected with CCNG2 or empty vector were implanted subcutaneously in the left flank of nude mice. Figure 7A shows the difference in the volume of tumors after 42 days. CCNG2 overexpression significantly reduces the proliferation of cells in tumors (Figure 7B). Furthermore, the number of apoptotic cells, as determined by a TUNEL assay, was increased in tumor cells overexpressing CCNG2 (Figure 7C). Ki67 is used as a marker of cell proliferation as it is absent in resting cells (27). The number of proliferating cells detected by a K167 immunohistochemistry assay was lower in tissue overexpressing CCNG2 (Figure 7D). These results indicate that glioma cells overexpressing CCNG2 can effectively prevent tumor formation *in vivo*.

## Discussion

Cyclin regulation and the PI3K/AKT signaling pathways are increasingly associated with cell proliferation in various cancers (28–30). Among the high-grade gliomas, the grade IV astrocytic tumor known as glioblastomas (GBM) is the most common, aggressive, and deadly tumor. So we chose four GBM cell lines (U138, U251, A172, and T98G) to use. In this study, we found that overexpressing CCNG2 suppresses proliferation of glioma cells, arrests the glioma cell cycle, and promotes glioma apoptosis. Furthermore, inhibition of AKT kinase increased the expression of CCNG2 and also suppresses the proliferation of glioma cells. Our results support accumulating evidence that the expression of CCNG2 diminishes as cancer progresses, with the lowest expression of CCNG2 associated with a more advanced stage of astrocytoma. A low level of CCNG2 is often associated with poor prognosis and a high recurrence rate such as in the advanced stages of leukemia and in high-grade oral, gastric, and thyroid cancers (19, 31–33). Moreover, in CCNG2-negative gastric adenocarcinoma and gastric carcinoma, patients had a lower 5-year overall survival rate than those who were CCNG2-positive (20). In addition, a study on the interaction of miR-1246 with CCNG2 in pancreatic cancer found that a high expression of miR-1246 was correlated with a worse prognosis and that CCNG2 expression was significantly lower in those patients (34).

We found that cells overexpressing CCNG2 were more likely to be in G0/G1 than control cells suggesting that CCNG2 can influence cell cycle arrest. It has been proposed that the CCNG2 protein co-localizes at centrosomes with phosphatase 2A (PP2A) and that alterations in centrosomal components could induce cell cycle arrest (35). When CCNG2 and PP2A form a protein dimer in cells, they are thought to inhibit cyclin-dependent kinase 2 and thus bind with the centrosome (16). Increasing levels of CCNG2 could modulate the cellular division processes through PP2A (36).

In our study, a reciprocal expression change occurred between CCNG2 and activated-AKT. High levels of activated-AKT corresponded to low expression levels of CCNG2 and *vice versa*. Disruptions in the PI3K/AKT pathway occur in 40% of all types of tumor (37). AKT is a serine/threonine kinase involved in the G1-S checkpoint transition and regulates cell cycle exit through an interaction with p21 (38). When activated, AKT phosphorylates cell cycle inhibitors, p21Cip1 and p27Kip1. The inactivation of these proteins promotes the cell cycle to shift from G1 to the S phase (39). Activating mutations in AKT1/2 results in accelerated tumorigenesis, however, AKT1 has an inhibitory effect on tumor cell invasion and migration, whereas AKT2 promotes metastasis (40). Although no direct evidence could be presented in the current study of a direct interaction between CCNG2 and AKT, a previous study has also reported that AKT phosphorylation negatively regulates CCNG2 protein levels (41), indicating a close association between CCNG2 and the AKT pathway. Thus, it is valid to hypothesize that CCNG2 may be influenced by a novel signaling pathway, which arrests the cell cycle *via* AKT kinase activation. A possible candidate could be the Nodal signaling pathway. Nodal, a member of the transforming growth factor-β superfamily, was found to interact with FoxO3a, which forms a complex with SMAD proteins to inhibit AKT activity (42). Nodal also interacts with FoxO3a to stimulate the CCNG2 promoter. It is thought that Nodal signaling promotes CCNG2 transcription by upregulating FoxO3a expression and inhibiting FoxO3a phosphorylation (42). It may be rewarding to further investigate the exact mechanism involved in the molecular interactions that regulate CCNG2 and AKT as they could have potential as therapeutic agents. Indeed, the AKT inhibitor MK-2206 has already been used in clinical trials, but it has limited anti-tumor activity as a monotherapy and is currently undergoing combination trials (43).

## Conclusion

Gliomagenesis is a multi-step process, which involves functional alteration of cell cycle regulatory members. CCNG2 has been confirmed as an inhibitory factor during carcinogenesis, whereas the AKT pathway is upregulated during carcinogenesis. In this study, we attempted to decipher the molecular characterization and functional consequences of CCNG2 during the dysregulation of the glioma cell cycle. Based on our data, we believe that the regulation of CCNG2, possibly by the AKT pathway, has potential in the management of astrocytomas.

## Author Contributions

LH and JW designed research, analyzed data, edited and revised the manuscript, revised and approved the final version of the manuscript. DZ, CW, and ZL performed the experiments and drafted the article. YL, DD, KH, LL, and YL helped to perform the research, contributed new reagents/analytic tools, and analyzed data.

## Conflict of Interest Statement

The authors declare that the research was conducted in the absence of any commercial or financial relationships that could be construed as a potential conflict of interest.
